# Characterization of autofluorescence and quantitative protoporphyrin IX biomarkers for optical spectroscopy-guided glioma surgery

**DOI:** 10.1038/s41598-021-99228-6

**Published:** 2021-10-08

**Authors:** David Black, Sadahiro Kaneko, Anna Walke, Simone König, Walter Stummer, Eric Suero Molina

**Affiliations:** 1 Department of Neurosurgery, University Hospital of Münster, Albert-Schweitzer-Campus 1, A1, 48149 Münster, Germany; 2 Carl Zeiss Meditec AG, Oberkochen, Germany; 3 University of British Columbia, Vancouver, Canada; 4 Department of Neurosurgery, Hokkaido University Graduate School of Medicine, Sapporo, Japan; 5 Core Unit Proteomics, Interdisciplinary Center for Clinical Research, University of Münster, Münster, Germany

**Keywords:** Blood-brain barrier, Glial biology

## Abstract

5-Aminolevulinic acid (5-ALA)-mediated fluorescence does not effectively depict low grade gliomas (LGG) or the infiltrative tumor portion of high-grade gliomas (HGG). While spectroscopy improves sensitivity and precision, this is currently limited by autofluorescence and a second protoporphyrin IX (PpIX) fluorescence state at 620 nm. We investigated the autofluorescence to better characterize the present spectra and thus increase PpIX quantification precision and sensitivity. This study included 128 patients undergoing surgery for malignant glioma. 5-ALA (Gliolan) was administered before anesthesia, and fluorescence was measured using a hyperspectral device. It was found that all 2692 measured spectra consisted of contributions from 620 to 634 nm PpIX, NADH, lipofuscin, and flavins. The basis spectra were characterized and their use in spectral unmixing led to 82.4% lower fitting error for weakly fluorescing areas (*p* < 0.001), and 92.3% fewer false positive tumor identifications in control measurements (*p* = 0.0065) compared to previous works. They also decreased the PpIX_620_ contribution, thus halving the mean Ratio_620/634_ (*p* < 0.001). The ratio was approximately 0 for HGGs and increasing for LGGs, as demonstrated previously. Additionally, the Ratio_620/634_, the MIB-1/Ki-67 proliferation index, and the PpIX peak blue-shift were found to be significantly related to WHO grade, fluorescence visibility, and PpIX contribution (*p* < 0.001), and the value of these three as quantitative biomarkers is discussed.

## Introduction

Fluorescence guided-surgery mediated by 5-Aminolevulinic Acid (5-ALA)-induced protoporphyrin IX (PpIX) fluorescence is a useful surgical adjunct in the treatment of malignant glioma. Fluorescence imaging aids in depicting malignant glioma tissue in real-time during surgery. 5-ALA is an naturally occurring metabolite and a non-fluorescent prodrug which takes part in the biosynthesis pathway of hemoglobin in humans. Malignant glioma selectively take up 5-ALA, converting this prodrug into PpIX for reasons to date not fully understood^1^. 5-ALA is administered in a dose of 20 mg/kg b.w. orally 4 h before induction of anesthesia. PpIX fluorescence can be visualized with commercially available microscopes equipped with special filter systems^2^. However, the sensitivity of this method is still not high enough to confidently discriminate between the solid and the infiltrative (low cellularity) component in malignant gliomas. Prognosis of patients harboring malignant glioma can be correlated with the extent of resection of tumor tissue^3,4^. Still, infiltrative tumor cells remain very difficult to detect during surgery, a reason why recurrences often occur within less than 2 cm of the original tumor margin^5^. Thus, surgical adjuncts such as fluorescence-guidance^6^ are used in malignant glioma surgery to maximize the extent of resection. In addition to its utility as a photosensitizer agent in photodynamic therapy (PDT)^7^, 5-ALA, (Gliolan, medac, Germany), a natural heme precursor, is the most established fluorophore in high-grade glioma (HGG) surgery. 5-ALA elicits the expression of fluorescent protoporphyrin IX in HGG cells, which naturally exists in humans and can be intraoperatively visualized with the assistance of commercially available filter systems^8–10^. PpIX exhibits maximum absorption of 405 nm blue light, the Soret Band, which elevates the molecule into an excited state from which it can either react with oxygen to create singlet oxygen that is cytotoxic and thus useful in PDT^11^, or it can fluoresce. Fluorescence occurs in a characteristic double emission peak at around 635 nm and 705 nm. The difference in wavelength between absorption and emission is called the Stokes shift and allows the excitation light and PpIX fluorescence to be separated using optical filters for visualization of tumors^12^.

However, only about 20% of LGGs display visible PpIX fluorescence during surgery^13^, and low density, infiltrative regions of HGGs also often do not fluoresce visibly^14^. Thus, fluorescence spectroscopy systems are being developed by a number of groups^14–16^ [Kaneko et al. accepted JNS]. In these devices, the emission spectrum is measured at selected points. After normalization for system parameters as well as inhomogeneous scattering and absorption across the tissue^17^, the relative PpIX fluorescence intensity is extracted using a spectral unmixing routine with known basis spectra^18^. In spectral unmixing, the optimal linear combination of the basis spectra minimizes the error between the measured and fitted spectrum, thus deriving a relative intensity, or relative concentration, for each present fluorophore including PpIX. PpIX fluorescence intensity is proportional to tumor cell density^19,20^ so it can be used to locate the tumor and help identify its density and thus malignancy. Such measurement systems greatly increase sensitivity^21^ as they often allow PpIX overexpression to be measured even when a tumor is not visibly fluorescing.

In LGGs, however, sensitivity is still limited to only 45%^22^. The same is likely true for low density regions of HGG. Noise in low-fluorescence measurements, and a high rate of false positive identification of PpIX signals leads to relatively poor delineation of tumor margins. These problems in detection are due in part to the oversimplified nature of the spectral unmixing algorithm. The PpIX fluorescence spectrum is dependent on the chemical microenvironment and the local pH^23–25^ which is due to the formation of different PPIX aggregation states. At acidic to neutral pH it was detected in assemblies of twice the size of those seen at alkaline pH^25^. Moreover, at high pH values aggregates are deprotonated and fluorescent. Protonated forms are nonfluorescent, but aggregates can encompass fluorescent PPIX molecules. As a result, in brain tissue, two fluorescing states of PpIX are observed: one that peaks at 634 nm, PpIX_634_, and another that peaks at 620 nm, PpIX_620_. Their relative intensities vary in different tumor types, densities, and malignancy levels^16,26–28^. The second peak has also been described in other biological context^29^ and in vitro^30^. It is presently assumed based on the available data that in low cellularity, alkaline and/or infiltrative zone tumor tissue predominantly PpIX_620_ dominates, whereas in high cellularity, acidic, HGG tissue PpIX_634_ is more intense^16,26^. This is the reason why presumably in low cellularity or low-graded portions of gliomas the PpIX_620_ state dominates.

To date, most spectroscopic measurements do not reflect the spectral contributions of PpIX_620_, since they assume that only PpIX_634_ is relevant in tumor tissues. Fortunately, it is relatively easy to rectify this problem by including separate basis spectra for PpIX_620_ and PpIX_634_. However, it becomes difficult to discuss PpIX concentration per se as it is unclear what combination of the two main molecular PpIX forms constitutes the overall PpIX “concentration”. Montcel et al. proposed a ratio between the two states’ intensities, Ratio_620/634_, which is related to the World Health Organization (WHO) classification^31^ and density of the measured region and can potentially be used as a biomarker in addition to PpIX intensity^16,26^.

In addition, the basis spectra used in the unmixing have a profound effect on the resulting PpIX quantification. There are a number of endogenous fluorophores in the brain which fluoresce in the same spectral region as PpIX, making it more difficult to isolate the PpIX signal. The emission spectra of these autofluorescence sources must be considered in spectral unmixing for best results. In their analysis, Montcel et al.^16^ used NADH, which is modeled as a decaying exponential that is fit to each individual measurement. In later work^26^, they added an approximate lipofuscin spectrum, modeled as a simple Gaussian curve^15^. However, by fitting an exponential curve for NADH directly to each measurement the actual shape of the NADH spectrum, not just its magnitude, is inconsistent. In reality, the emission spectrum should be fixed and should only vary in magnitude. Similarly, the Gaussian lipofuscin curve is highly simplified. Mehidine et al. also used simplified spectra, including NADH, tryptophan, tyrosine, and collagen^32^. Finally, there are other fluorophores present in the brain, including elastin, pyridoxine, flavins, and both free and protein-bound NADH^33^, which, as far as we know have not been considered in spectral unmixing so far.

While including too many basis spectra can lead to overfitting, especially if they are not needed, the spectral unmixing routine theoretically simply assigns a magnitude of 0 to any spectrum not present in a measurement, ignoring noise and measurement artefacts. On the other hand, if a spectral component is present but not provided as a basis spectrum for the unmixing, the unmixing algorithm is forced to compensate by artificially inflating the contribution of other components. This is observable through large fitting errors in the unmixing and can for example artificially increase one or both PpIX components, thus affecting the Ratio_620/634_ and leading to false positive PpIX signal interpretation.

In this study, we characterized the spectra of the fluorophores present in the measured fluorescence emission spectra more precisely in order to improve the sensitivity and precision of PpIX quantification.

## Methods

Patients undergoing surgery for malignant glioma at the University Hospital of Münster were included in this study. Indication for surgery was discussed in an interdisciplinary tumor board. Biopsies (n = 275) of tumor bulk from 128 patients were obtained intraoperatively and were measured immediately using an ex vivo hyperspectral device, after minimal light exposure to reduce photobleaching. Of these, 12 were control samples: GBM where no 5-ALA was administered, e.g. in an emergency situation, or tissue from epilepsy procedures, where temporal lobe resections were indicated. Approximately 10 fluorescence spectra were extracted from different regions of each tissue, depending on the size of the sample. In total, this study used 2572 measured fluorescence spectra and 120 control measurements.

Informed consent was obtained from each individual in this patient collective. All procedures performed in studies were in accordance with the ethical standards of the institutional and/or national research committee and with the 1964 Helsinki declaration and its later amendments or comparable ethical standards. Data collection and scientific use of biopsies had previously been approved by the ethics committee of the University of Münster.

**Figure 1:**
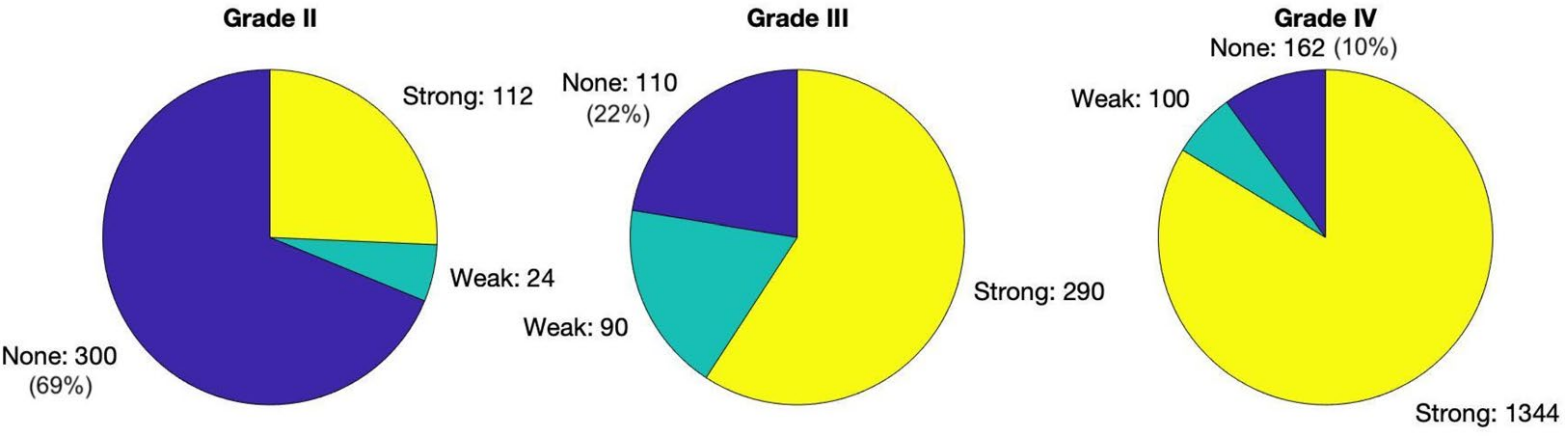
Fluorescence visibility during surgery versus WHO Grade. The WHO Grade I tissues all showed no visible fluorescence. Fluorescence categorized as strong (yellow), weak (green), not visible (blue). The number of measured tissues is given for each response.

Autofluorescence spectra were derived from the measurements by analyzing spectral features consistently present in the data that were independent from other features such as glioma-derived PpIX fluorescence, and comparing them to known fluorophore data available in the literature. The contributions of the PpIX_620_ state, [PpIX_620_], PpIX_634_ state, [PpIX_634_], and their ratio, Ratio_620/634_ were analyzed and correlated with pathological parameters including WHO grade, MIB-1/Ki-67 proliferation index, and fluorescence visibility. The latter was assessed by the surgeons as ‘none’, ‘weak’, or ‘strong’ for every biopsy. The breakdown of fluorescence visibility vs. WHO grade is shown in Fig. 1.

### Data preprocessing

A hyperspectral measurement device built on a Zeiss OPMI Pico microscope was used to extract spectral information from the fluorescing samples, ex-vivo as described previously^18^. Spectra were extracted from selected pixels of interest and were corrected for a number of parameters specific to the measurement system such as the camera’s wavelength-dependent sensitivity. They were then normalized according to the two-peak routine proposed by Valdes et al.^17^ using white-light data. This normalization corrects for inhomogeneous scattering and absorption coefficients across the tissue surface.

The measurements were then decomposed into their spectral constituents, using the known emission spectra of the major sources of fluorescence in the tissue in order to evaluate how much of each component was present. These sources included PpIX as well as autofluorescence, and their spectra form a basis which spans the space of possible fluorescence measurements. To perform the ‘spectral unmixing’, we projected the measured curve onto the basis spectra to find the contribution of each using a least squares optimization routine.

Based on experiments with reference samples (so-called phantoms) of known PpIX concentration (µg/ml), the contributions of the PpIX spectra can be mapped to absolute PpIX concentration in µg/ml. We created phantoms as described by Valdes et al.^17^ to characterize the measurement system. However, as a result of their composition, the phantoms generally contained only a single PpIX fluorescence state. They lacked most of the autofluorescence sources present in the brain, and a linear correlation of PpIX intensity and concentration was not possible^24,34^. Moreover, there is large variation between measurement devices. Hence, the calculated concentrations in this study are not intended for comparison with studies from other groups, or known values, but are self-consistent and provide a useful way to compare the relative intensities of the various spectra present in the measured light. They will be referred to as ‘contributions’ not ‘concentrations’ to avoid misinterpretation. Spectral contributions are written as [PpIX_6xx_] and are given in arbitrary units (a.u.), where the units stem from the normalization procedure described above.

It is important to note that all measurements presented in this paper were carried out on the same device, so the scaling and the measurement system characteristics, artefacts, and imperfections were consistent throughout the study. In addition, any scaling difference between methods and devices should cancel out when finding the [PpIX_620_]/[PpIX_634_] ratio, so that the Ratio_620/634_ results can indeed be compared between groups.

### Fluorescence model

In order to discuss the PpIX contributions and the Ratio_620/634_ further, it is essential to have precisely characterized all basis spectra. The PpIX_620_ and PpIX_634_ spectra are well known and available in the literature^15–17,23,24,26,35^. Their shapes were obtained by phantom measurement. However, there is less clarity about the molecular contributions to autofluorescence. Possible candidates include elastin, collagen, pyridoxine, tryptophan, flavins, lipo-pigments, and both free and protein-bound NADH^33^. The first four on this list do not fluoresce in the same spectral range as PpIX and are of little interest when studying PpIX. However, NADH is apparent in the data and its exponentially decaying tail overlaps with PpIX^16,26^.

**Figure 2:**
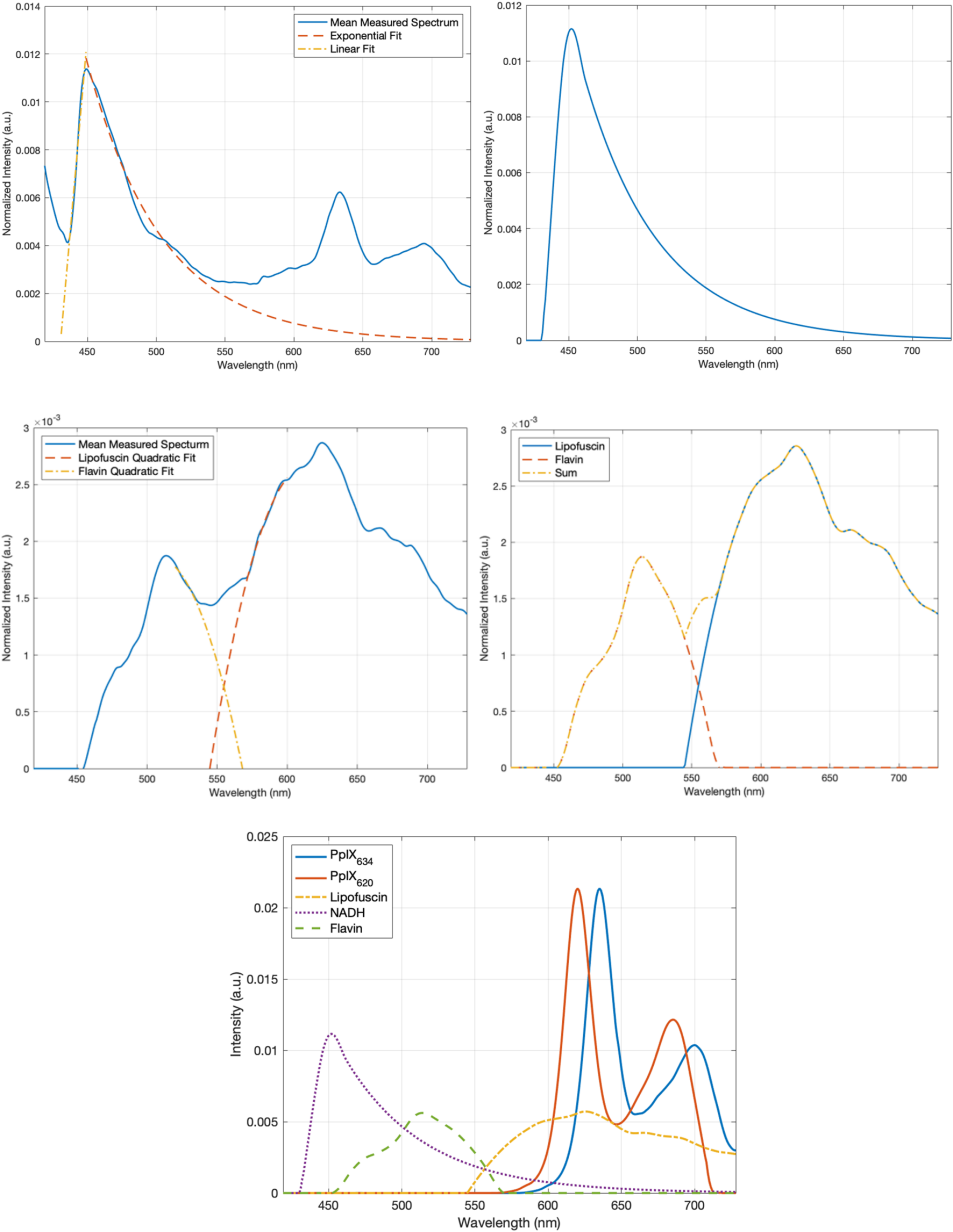
(Top) Mean spectrum containing the NADH spectral contribution with the expected NADH curves fitted (left) and the resultant NADH basis spectrum (right). (Middle) Lipofuscin and flavin spectra fitted to the mean measured spectrum (left) and the resulting basis spectra and their sum (right) which closely matches the mean measured spectrum. (Bottom) The resulting 5 basis spectra from this analysis.

The NADH spectrum was characterized as follows:

1. Spectra (n = 248) with a large NADH component, where the NADH peak magnitude was greater than the PPIX peak, were selected.
2. The spectra were normalized at the NADH peak so that they all had an NADH peak of the same magnitude.
3. The 248 spectra were averaged to find a mean spectrum.
4. An exponential decay curve^15^ was fit to the section from 450 to 525 nm and a linear curve from 430 to 450 nm as illustrated in Fig. 2 using the ‘fit’ function in MATLAB (Mathworks Inc.).
5. The two curves were concatenated and smoothed using Savitzky-Golay filtering to create the smooth approximate NADH spectrum without losing details (Fig. 2).

The next spectrum, lipofuscin, is of primary interest because it overlaps completely with the PpIX fluorescence and thus biases the calculations for the PpIX contribution. Previous studies of PpIX and the Ratio_620/634_ for neurosurgery^16,26^ have mostly neglected this spectrum, but we found it plays a substantial role in brain fluorescence measurements. To characterize the spectrum, the same procedure was followed as for NADH, but using only the 12 control tissues without PpIX. These tissues constituted normal brain from epilepsy patients (n = 6, histological reactive brain changes without tumor cells) and glioblastoma patients where no 5-ALA was given (n = 6, histological WHO grade IV glioblastoma). Additionally, the NADH spectrum was fitted to the measured control spectra in the NADH peak region in order to remove the NADH portion from the spectra and to isolate the remaining fluorophores. When the resulting curves were averaged, it became apparent that there were two fluorophores present (Fig. 2). The lipofuscin spectrum was augmented by an extra peak at ~ 512 nm, which likely originated from flavins^36–38^. Quadratic curves were fitted to both the lipofuscin and the flavin peaks to match the expected shapes of the spectra (Fig. 2, middle left). These were combined with the separated averaged spectrum and smoothed to create a lipofuscin and a flavin spectrum, respectively (Fig. 2, middle right).

Thus, we arrived at five basis spectra: PpIX_620_, PpIX_634_ and the three autofluorescence spectra for lipofuscin, NADH, and flavin. Different combinations of these five spectra (Fig. 2 bottom) constituted every measured spectrum. This was validated as described in the Results section. We could therefore spectrally unmix all the measured, normalized spectra to obtain the desired PpIX contributions [PpIX_620_] and [PpIX_634_].

### Statistical analysis

Calculations were performed using the commercially available software MATLAB (The Math Works Inc., Natick, MA). *p*-values were obtained with the non-parametric Wilcoxon Rank Sum Test as the distributions were either discrete or highly skewed. A *p*-value < 0.05 was considered statistically significant. All reported *p*-values are two-tailed.

## Results

### Fluorescence model

**Figure 3:**
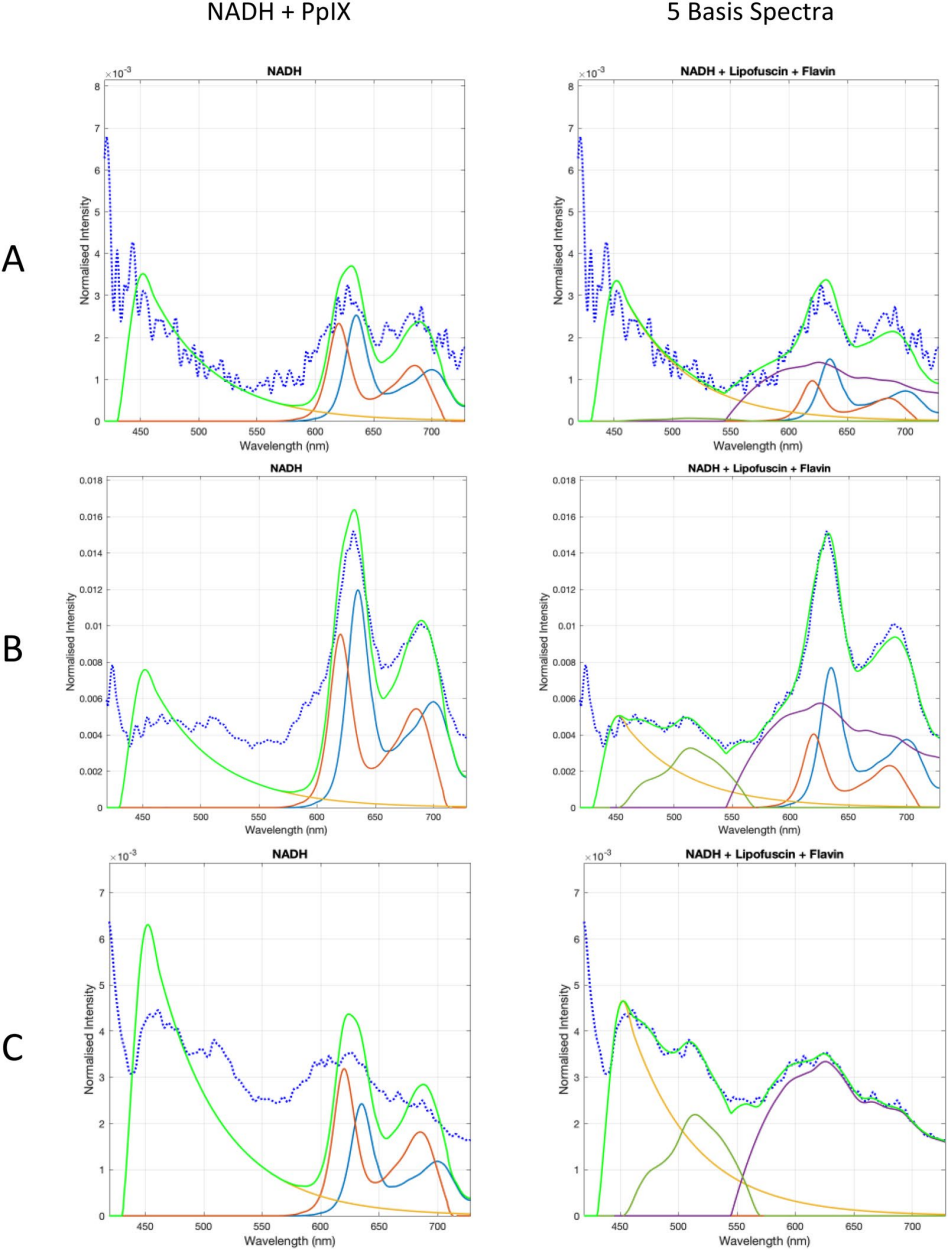
Unmixing of three spectra (**A**, **B**, **C**), each using the five basis spectra (right) and just NADH (left) (orange: PpIX_620_, blue: PpIX_634_, purple: lipofuscin, yellow: NADH, green: flavin). The measured spectrum is shown in blue dots, and the fitted curve is bright green. As was the case with all spectra, the fit was better using the five basis spectra than with just NADH. Spectrum C is the control without PpIX showing how the five basis spectra eliminate false positives.

A total of 2572 fluorescent and 120 control measurements were obtained intraoperatively. First, all 2692 spectra were unmixed and checked visually, both using all determined basis spectra (Fig. 2 bottom), and using only the PpIX and NADH spectra, as performed previously^16^, for subsequent comparison. Qualitatively, using the five basis spectra, the fit was significantly improved and more realistic with not a single exception among all samples. Three representative examples are shown in Fig. 3. For quantitative estimation of the improvement, the error between the fit and the measured spectum was calculated using Eq. (1) for every spectrum, where $\Phi_{meas}$ is the measurement and $\Phi_{fit}$ the fit generated by the unmixing.

1 $$e = \left| {\Phi_{meas} - \Phi_{fit} } \right|^{2}$$

Of course, in strongly fluorescing HGG spectra, which consisted predominantly of PpIX_634_, the use of the additional basis spectra made little difference. However, in weaker spectra, with [PpIX] < 1, the use of the five basis spectra decreased the error approximately six-fold. Even when considering all spectra, the error was 50% greater when using only NADH. One could argue that only the fit in the PpIX fluorescence region (600 nm to 730 nm) matters since the PpIX contribution is used to recognize tumors. Considering only this region, the error was still approximately halved using the five basis spectra. These results are summarized in Table 1, and show clear improvement in the effectiveness of the spectral unmixing. Some examples are shown in Fig. 3, and the results are further investigated in the Discussion section.

**Table 1:** Mean fit error between measured and unmixed spectra (a.u.) from various groups, and over either the whole spectrum (420 nm to 730 nm) or just the PpIX region (600 nm to 730 nm).

	NADH	NADH + Lipofuscin + Flavin	*p*-value
Error (only PpIX region), ([PpIX] < 1) (a.u.)	2.0475 ± 0.0508	1.1126 ± 0.0373	< 0.001
Error ([PpIX] < 1) (a.u.)	37.716 ± 1.8116	6.6350 ± 0.2742	< 0.001
Error (LGGs) (a.u.)	53.1293 ± 3.0779	19.1896 ± 1.7461	< 0.001
Error (All spectra) (a.u.)	214.5525 ± 6.504	150.0299 ± 5.8391	< 0.001

For the neurosurgery application, another important parameter is the rate of false positive PpIX signals, where the system identifies nonzero PpIX contributions in measurements where no PpIX is present. Using the five basis spectra during spectral unmixing, the rate of false positives in the control measurements fell from 97.5% (using only NADH and PpIX) to 7.5% (using five spectra) with a cutoff of 0.001 a.u. below which contributions were set to zero as the signal to noise ratio approached 1. This constitutes a 90% increase in sensitivity (true positive rate) (*p* = 0.0065). The mean of the calculated [PpIX_620_] and [PpIX_634_] of the control spectra was 0.0078 a.u. using just NADH, and 0.0007 a.u. using the five basis spectra (*p* = 0.015). This is an order of magnitude improvement in specificity (true negative rate).

### PpIX results

Having found more realistic basis spectra, it was then possible to explore the PpIX-related results of the measurements including the contributions of each PpIX state, their ratio, and the relation of these values to fluorescence visibility, WHO grade, and proliferation index. In particular, the Ratio_620/634_ results was compared to the results of Alston, Montcel, et al.^16,26^. The primary results are summarized in Table 2.

**Table 2:** Tissue overview, categorized by fluorescence visibility and WHO grade (I, II = LGG; III, IV = HGG).

Fluorescence visibility	# of Samples	Mean [PpIX_620_]	Mean [PpIX_634_]	Mean ratio [PpIX_620_]/ [PpIX_634_]	Proliferation index (Ki-67)
None	612	0.0542 ± 0.0023	0.2517 ± 0.0145	0.4004 ± 0.0490	17.436 ± 0.591
Weak	214	0.1556 ± 0.0129	2.2270 ± 0.1386	0.1185 ± 0.0107	29.327 ± 1.191
Strong	1746	0.2262 ± 0.0127	10.5886 ± 0.2699	0.0366 ± 0.0016	24.118 ± 0.289
*p*-value	–	< 0.001	< 0.001	< 0.001	< 0.001
**WHO grade**					
I	40	0.0018 ± 0.0006	0.0153 ± 0.00019	0.0799 ± 0.0256	1.00 ± 0.000
II	436	0.1281 ± 0.01456	1.4830 ± 0.1320	0.4303 ± 0.0669	6.00 ± 0.146
III	490	0.1000 ± 0.0085	3.8423 ± 0.2220	0.1145 ± 0.0108	18.514 ± 0.386
IV	1606	0.2227 ± 0.0132	10.3627 ± 0.2952	0.0487 ± 0.0022	28.871 ± 0.304
*p*-value		< 0.001	< 0.001	0.007	< 0.001

The given uncertainty is the standard error of the mean. Units are a.u.

The relations between Ratio_620/634_ and both WHO grade and fluorescence visibility were significant with > 99.9% confidence. As tumor malignancy increased and thus, for the most part, fluorescence visibility increased (Fig. 1), the 634 nm PpIX state became more dominant compared to PpIX_620_. Both [PpIX_620_] and [PpIX_634_] had a positive correlation to WHO grade and fluorescence visibility, but [PpIX_634_] grew more quickly, leading to the decreasing ratio.

**Figure 4:**
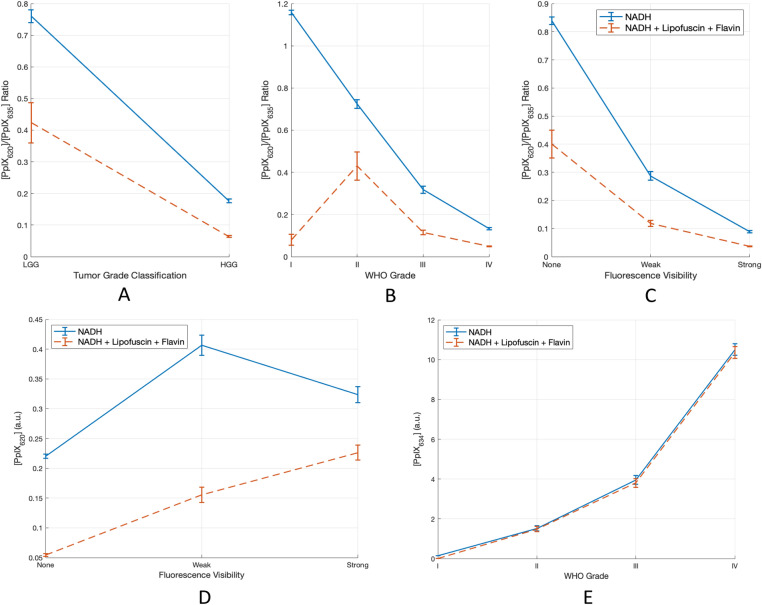
(**A**, **B**, **C**) Ratio_620/634_, (**D**) [PpIX_620_], and (**E**) [PpIX_634_] vs. WHO Grade and fluorescence visibility, using unmixing with only NADH and PpIX, and with all five basis spectra. The results from using only NADH show greatly inflated [PpIX_620_], and thus artificially high Ratio_620/634_.

A comparison of these results with those obtained when using only NADH as the autofluorescence basis spectrum is presented in Fig. 4. The lack of the lipofuscin spectrum inflated the [PpIX_620_] values to 2–5 times their expected value while having little effect on [PpIX_634_]. As a result, the Ratio_620/634_ values were approximately twice as high as they should be. The data are compared previous work in the Discussion section, where the anomalously low ratio at WHO grade I is also discussed.

### Proliferation index

The proliferation index, Ki-67 (also known as MIB-1), correlated significantly with WHO grade and fluorescence visibility (*p* < 0.001). As expected, it has a significant (*p* < 0.001) correlation with Ratio_620/634_. However, this relationship did not appear to be linear. A line was fitted to the log of the data, with Pearson product-moment correlation *r* = − 0.514, indicating a medium-strong linear correlation in log-space. The slope of the line of best fit was − 0.35 indicating the approximate functional relationship shown in Eq. (2).

2 $$\left( {\text{Ki - 67}} \right) \propto \left( {Ratio_{{\frac{620}{{634}}}} } \right)^{{ - \frac{1}{3}}}$$

### PpIX blue shift

**Figure 5:**
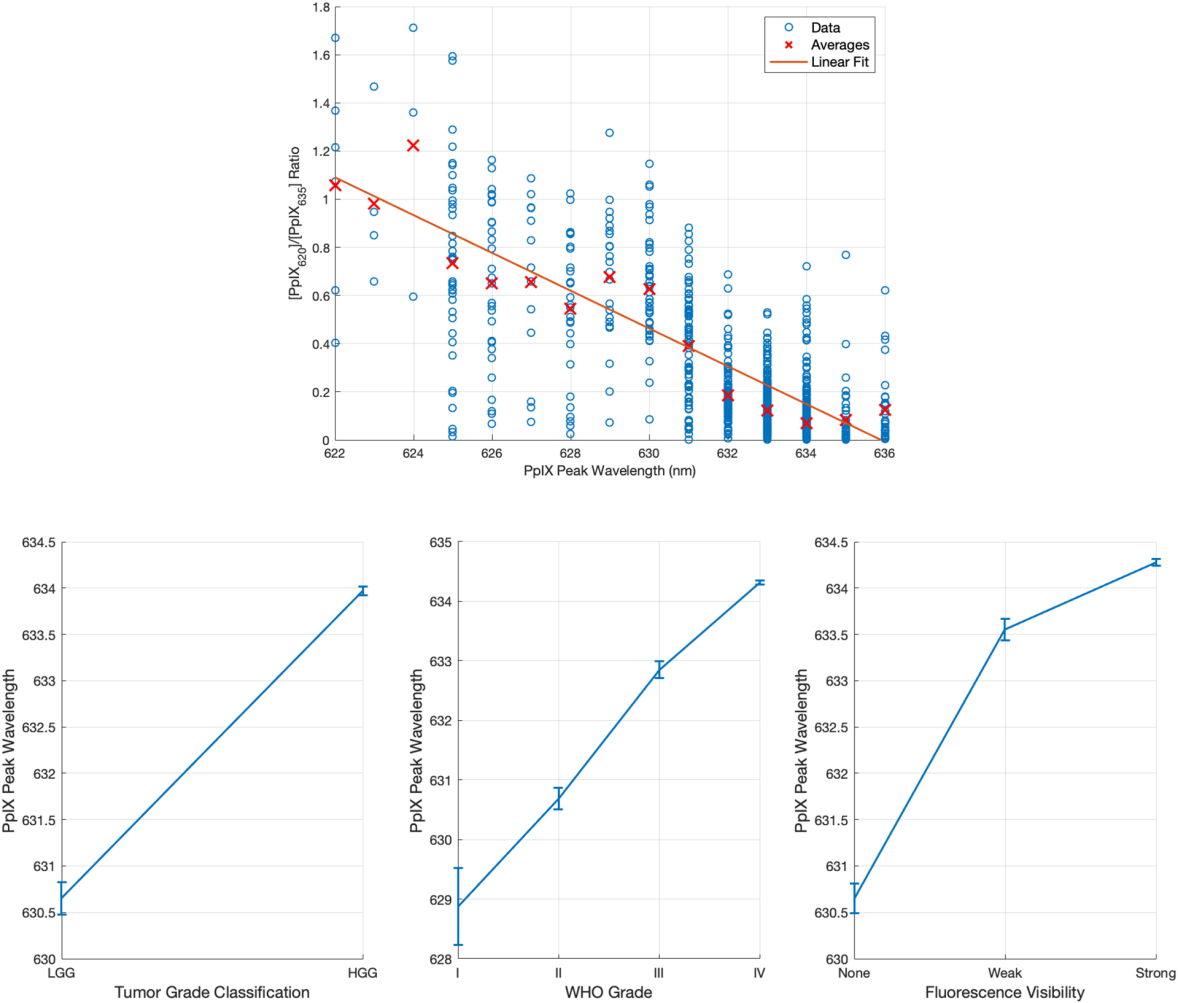
(Top) Ratio_620/634_ versus PpIX peak wavelength. The Pearson correlation coefficient is − 0.76, indicating a strong linear correlation. (Bottom) PpIX peak wavelength versus WHO grade, tumor class, and fluorescence visibility, showing strong correlation with each.

As the Ratio_620/634_ increased, the location of the main PpIX peak of the measured spectrum shifted from 634 nm towards 620 nm as illustrated in Fig. 5. There was a strong correlation between Ratio_620/634_ and the PpIX peak wavelength with a Pearson correlation coefficient of -0.76. The PpIX peak position was also significantly related to [PpIX_620_], [PpIX_634_], Ki-67, fluorescence visibility, and WHO grade, all with *p* < 0.001. The latter two are plotted in Fig. 5 (bottom). The value of this potential biomarker is discussed in the Discussion section.

## Discussion

In this manuscript we improve the common analysis routine of fluorescence spectra by further characterization of autofluorescence spectra, taking into account not only the contribution of NADH, but also that of lipofuscin and flavins. The newly generated basis spectra matched the data better and led to more accurate spectral unmixing and far lower false positive rates in a diverse population of 128 patients. Using these basis spectra, 2692 measured fluorescence spectra from the 128 patients were spectrally unmixed and the [PpIX_620_], [PpIX_634_], and resulting Ratio_620/634_ were calculated. The results are presented above, and differ from values calculated previously in literature^16,26^.

Though this is an improved methodology, some caveats merit mention. Firstly, detecting very low PpIX concentrations is limited by the signal to noise ratio of the measurement system, as seen in Fig. 3A. Secondly, this method assumes that the fluorophores have a constant emission spectrum irrespective of possible interactions between them and with their microenvironment. For example, NADH exists in both free and protein-bound form^33^, and of course PPIX has the two distinct states which we model as two different fluorophores. There could be other such interactions that are not modeled here. We assume, however, that their effect is small. Further, it has been shown that PPIX fluorescence is present in healthy tissue as well^32,39^. Thus, the presented method of deriving the lipofuscin basis spectrum embeds this slight PPIX fluorescence in the lipofuscin spectrum. However, as we are interested in identifying tumor tissue, not precisely measuring PPIX concentration, this is not a problem. In fact, by leveraging the large amount of data used in this work, it is likely that the difference between healthy and tumor tissue is more effectively captured in this way than it would be by more precisely determining PpIX concentration and trying to impose a healthy tissue cut-off. While this lipofuscin spectrum would tend to underestimate PpIX concentrations, it does so equally for the two different PpIX states, and the magnitude of this underestimation is negligible. This can be seen in Fig. 3, looking at the small details of the lipofuscin basis spectrum which are possibly artefacts of the embedded PPIX signal, and comparing their magnitude to those of the actual PPIX spectra. Thus, the absolute PpIX concentrations are negligibly affected by this fact, and the presented ratios are unaffected.

### Spectral unmixing fit quality

The improved fit quality of the spectral unmixing seen in Table 1 is not the result of overfitting, because the chosen fluorophores are known to be present in brain tissue and their fluoresence was clearly present in the data. Although the data were obtained from a large and diverse patient cohort, the use of the five basis spectra consistently provided a very precise fit in every single measurement. In addition, as shown in the Results section, the rate of false positive PpIX signals was greatly reduced, which allowed more precise detection of tumor margins.

Finally, even if a spectrum contained only NADH and PpIX and the use of the two additional basis spectra for lipofuscin and flavin was therefore superfluous, the result of using them was never worse than using only NADH and PpIX. The spectral unmixing routine finds the mathematically optimal fit given the basis spectra, so it simply sets the contributions of the unneeded basis spectra to zero, so they have no effect. This is demonstrated in spectrum A of Fig. 3, where no detectable flavin autofluorescence was present.

### PpIX blue shift

The more PpIX_620_ is present relative to PpIX_634_ in a measurement, the more the peak shifts towards shorter wavelengths. The benefit of using this blue shift as a biomarker instead of the Ratio_620/634_ is that it is independent of the choice of autofluorescence basis spectra. Interestingly, the three plots in Fig. 5 also appear more linear than the Ratio_620/634_ curves in Fig. 4, and the WHO grade I tumors are not outliers as in Fig. 4B. Thus, the PpIX peak position could be a more robust and objective measure than Ratio_620/634_. However, it does not have the same dynamic range as the Ratio_620/634_ and therefore cannot resolve small differences in malignancy or tumor cell density. In addition, small variations in the PpIX peak due to noise can lead to changes in the detected peak position. Thus, noise-robust methods must be used to determine the peak. In this work, the signal was smoothed first using a moving mean filter before the peak was determined. This process is imperfect, though. An alternative would be to fit a PpIX like signal to the noisy one and determine its peak. However, this relies on knowing the PpIX basis spectrum precisely, which negates the primary benefit of this non-model-based method. The spectral unmixing method, on the other hand, is relatively immune to noise, except at very low fluorescence levels. Ultimately, further studies are needed before this can be used in practice.

### Comparison to the literature

Alston et al.^26^ measured the Ratio_620/634_ from solid and infiltrating parts of HGG and LGG tumors. While we did not differentiate between solid and infiltrative parts of tumors and only imaged the solid part, we still evaluated HGGs and LGGs, which have similar ratios to the low density infiltrative regions of HGGs^26^. Alston et al. found, as did we, that the ratio was close to 0 for HGGs and increased in LGGs. The results are compared in Table 3.

**Table 3:** Comparison of average Ratio_620/634_ values from literature to our results, with and without the lipofuscin and flavin autofluorescence basis spectra.

	Montcel et al.^16^	Alston et al.^26^	This study (5 basis spectra)	This study (3 basis spectra)
HGG solid tumor	0.03 ± 0.02	< 0.1	0.064 ± 0.003	0.18 ± 0.006
LGG solid tumor	1.25 to 1.62	1.6	0.42 ± 0.07	0.76 ± 0.02
HGG infiltration zone	1.04 ± 0.1	1.3	–	

While the HGG results were similar, differences can be explained by the slightly different NADH and PpIX spectra used. In addition, Montcel et al.’s HGGs consisted only of WHO grade IV while the present study included 490 grade III and 1604 grade IV measurements. The average value dropped to 0.048 ± 0.002 when considering only grade IV tumors.

Among the low-grade tumors a greater difference was observed. This is likely due to the large differences in [PpIX_620_], which was inflated when not using the five basis spectra, as seen in Fig. 4 (bottom left). Without the lipofuscin and flavin contributions, the mean Ratio_620/634_ more than doubled from 0.123 to 0.285. This accounts for some of the difference between the Montcel and Alston values and those found in the present study. Additionally, the differing measurement system and normalization would play a role. Finally, Alston et al. had only grade II tumors in their LGG group while we had 40 grade I measurements. Perhaps due to the extremely weak PpIX signal strength of these measurements and the small signal to noise ratio, the mean Ratio_620/634_ was anomalously low as seen in the top middle panel of Fig. 4. Because there were so many more grade II tumors than grade I, this had little effect on the mean LGG ratio; it nonetheless drags the ratio down slightly. Without the grade I tumors, the mean LGG Ratio_620/634_ was 0.43.

With a decrease in Ratio_620/634_ from grade II to I (Fig. 4B), the PPIX blue shift should decrease as well. However, the PPIX peak wavelength for grade I tumors in Fig. 5 may be artificially low because the small signal to noise ratio in the grade I tumors affects how accurately the PpIX peak location can be determined. Furthermore, in these extremely weak spectra, the lipofuscin begins to dominate the PPIX, which drives the apparent blue shift further towards 620 nm.

Finally, we did not use a photoproduct spectrum in this work because the tissues were removed and immediately imaged with minimal light exposure and thus minimal photobleaching. No photoproduct was detected in the measurements.

## Conclusion

This work aimed to add to the discussion in fluorescence guided neurosurgery, especially with regard to concepts introduced in previous articles^16,26^, and thus to increase the sensitivity and precision of PpIX quantification in fluorescence spectroscopy. It was found that improved basis spectra decreased the artificial inflation of [PpIX_620_], thus approximately halving the average Ratio_620/634_ values. The ratio was nonetheless close to 0 for HGGs and increasing for LGGs. Additionally, the proliferation index, Ki-67, significantly related to WHO grade, fluorescence visibility, PpIX contribution, and Ratio_620/634_. Finally, the blue shift of the peak wavelength of PpIX, showed potential as biomarker. Both this shift and the Ratio_620/634_ will have to be studied in more depth before they can serve as biomarkers. However, the use of the derived autofluorescence spectra in combination with the two PpIX fluorescence states in spectral unmixing routines offer promising results towards increased sensitivity and precision in PpIX quantification and delineation of tumor margins. Future work can also investigate if the other present fluorophores such as NADH, lipofuscin, and flavins have any predictive value in recognizing tumor tissue.
